# Multiple genetic loci define Ca^++^ utilization by bloodstream malaria parasites

**DOI:** 10.1186/s12864-018-5418-y

**Published:** 2019-01-16

**Authors:** Liana Apolis, Joanna Olivas, Prakash Srinivasan, Ambuj K. Kushwaha, Sanjay A. Desai

**Affiliations:** 1 The Laboratory of Malaria and Vector Research, National Institute of Allergy and Infectious Diseases, National Institutes of Health, Rockville, MD USA; 2 Florida State University College of Medicine, Tallahassee, Florida, USA; 3 Department Molecular Microbiology and Immunology, Johns Hopkins Malaria Research Institute, Baltimore, MD USA

**Keywords:** Malaria, Calcium, Antimalarial drug targets, Linkage analysis, EGTA, *Plasmodium falciparum*, Merozoite invasion

## Abstract

**Background:**

Bloodstream malaria parasites require Ca^++^ for their development, but the sites and mechanisms of Ca^++^ utilization are not well understood. We hypothesized that there may be differences in Ca^++^ uptake or utilization by genetically distinct lines of *P. falciparum*. These differences, if identified, may provide insights into molecular mechanisms.

**Results:**

Dose response studies with the Ca^++^ chelator EGTA (ethylene glycol-bis(β-aminoethyl ether)-N,N,N′,N′-tetraacetic acid) revealed stable differences in Ca^++^ requirement for six geographically divergent parasite lines used in previous genetic crosses, with the largest difference seen between the parents of the HB3 x Dd2 cross. Genetic mapping of Ca^++^ requirement yielded complex inheritance in 34 progeny clones with a single significant locus on chromosome 7 and possible contributions from other loci. Although encoded by a gene in the significant locus and a proposed Ca^++^ target, PfCRT (*P. falciparum* chloroquine resistance transporter), the primary determinant of clinical resistance to the antimalarial drug chloroquine, does not appear to contribute to this quantitative trait. Stage-specific application of extracellular EGTA also excluded determinants associated with merozoite egress and erythrocyte reinvasion.

**Conclusions:**

We have identified differences in Ca^++^ utilization amongst *P. falciparum* lines. These differences are under genetic regulation, segregating as a complex trait in genetic cross progeny. Ca^++^ uptake and utilization throughout the bloodstream asexual cycle of malaria parasites represents an unexplored target for therapeutic intervention.

## Background

Development of *Plasmodium* parasites within erythrocytes accounts for most of the clinical sequelae of malaria, which remains a leading infectious cause of morbidity and mortality worldwide. In the virulent human pathogen, *P. falciparum*, intraerythrocytic growth protects the parasite from host immune responses, but also limits the pathogen from access to nutrients and critical ions in host plasma.

Ca^++^ is one such critical ion because it is present at relatively high concentrations in plasma (~ 1.1 mM ionized and 1.4 mM bound to proteins [1]), but available at much lower levels within the erythrocyte (< 100 nM, [2, 3]). Multiple studies have determined that the intracellular parasite requires Ca^++^ [4–6] and that this pathogen may fulfil this need by increasing Ca^++^ permeability at the host membrane [7–11]. Interestingly, the plasmodial surface anion channel (PSAC), a broad selectivity nutrient and ion channel induced on the host erythrocyte membrane [12, 13], is not responsible for the increased Ca^++^ permeability [6]. In addition to roles in parasite development, Ca^++^ is also needed for egress from the host cell as determined through selective loading of membrane-permeable Ca^++^ chelators [14]. A separate requirement for the subsequent invasion of erythrocytes has also been identified [4, 15, 16].

Despite evidence for multiple distinct roles of Ca^++^ in the bloodstream malaria parasites, the molecular targets and mechanisms of calcium acquisition remain poorly characterized. Computational analyses of the parasite genome database have identified multiple genes encoding Ca^++^-binding proteins via either the EF-hand or C2 domains [17]. A single calmodulin gene, two calcineurin subunits, several centrins, and Ca^++^ dependent protein kinases have been identified as EF-hand domain containing proteins in *P. falciparum*; several C2 domain containing proteins have also been identified [18]. While these genes have been characterized to varying extents, few have been studies through biochemical studies to confirm regulated response to Ca^++^ binding. As these domains were identified in higher organisms [19, 20], it is possible that *Plasmodium spp.* use additional, uncharacterized protein motifs and Ca^++^ regulatory mechanisms. Thus, the molecular basis of Ca^++^ requirement and the roles of Ca^++^ signaling in malaria parasite biology remain unclear.

To explore the global effects of Ca^++^ and overcome the limitations of computational approaches, we reasoned that quantitative trait locus (QTL) mapping in parasite genetic crosses may provide an unbiased method of identifying the major Ca^++^ targets. This method has successfully identified parasite molecules involved in drug resistance, invasion, and nutrient uptake [21–24]. Here, we identified differences in Ca^++^ requirement for laboratory lines of *P. falciparum*. We then tracked inheritance of a significant difference in the Dd2 x HB3 genetic cross and used QTL mapping to identify possible Ca^++^ targets. Our studies implicate complex roles for Ca^++^ throughout the parasite cycle and suggest a genomic locus with a significant effect.

## Results

### *P. falciparum* lines exhibit differing susceptibilities to the Ca^++^ chelator EGTA

**Fig. 1:**
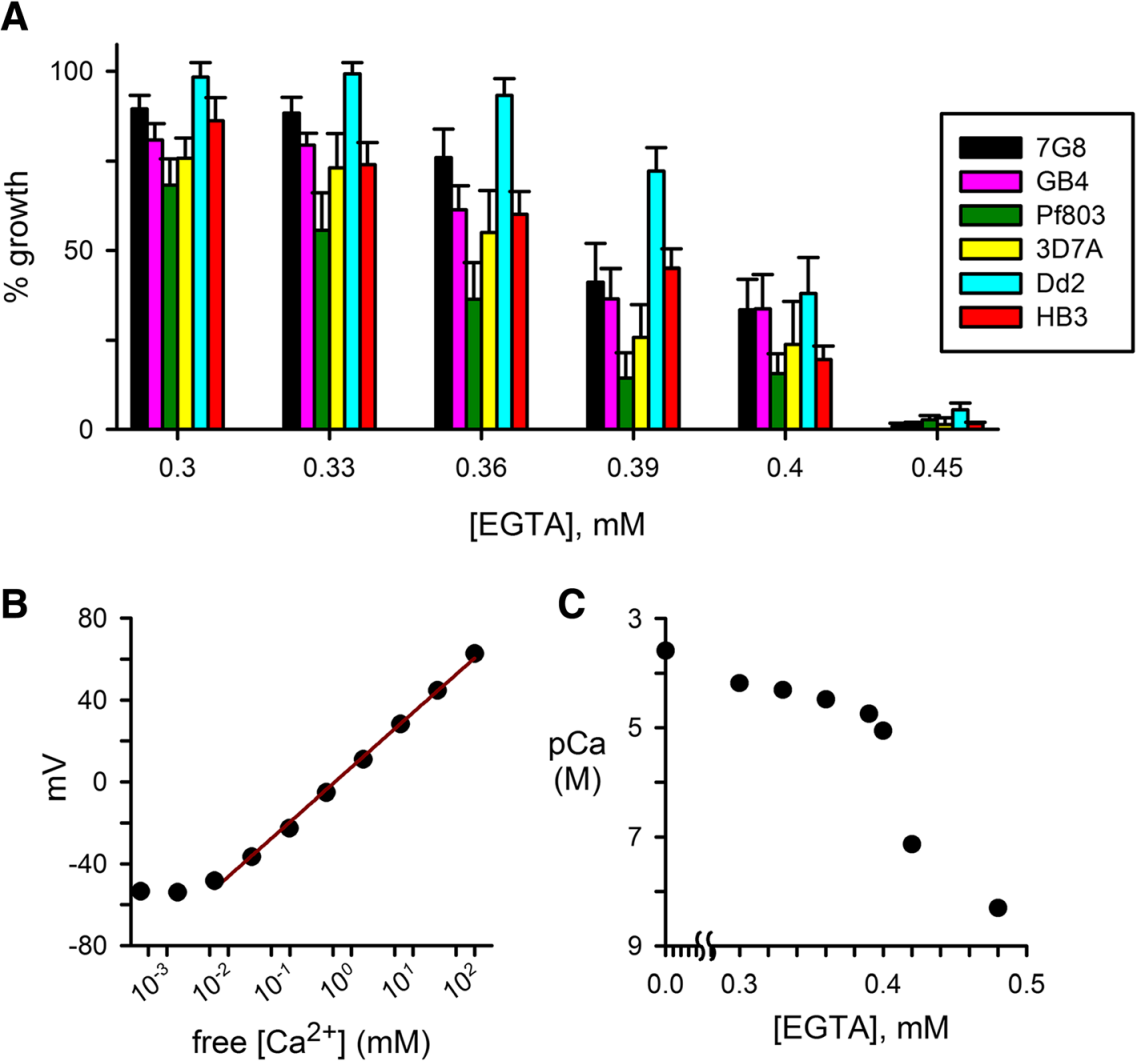
Differing Ca^++^ requirements for in vitro cultivation of common *P. falciparum* lines. **a** Mean ± S.E.M. growth of laboratory parasite lines in media supplemented with indicated EGTA concentrations, normalized to 100% growth for each parasite in media without EGTA. **b** Standard curve for free Ca^++^ measurements using ion-sensitive electrode. The solid line reflects a linear regression fit using data for Ca^++^ between 6 μM and 100 mM and corresponds to an electrode response of 26.7 mV/decade, in agreement with Nernstian predictions for a divalent cation. **c** Free Ca^++^ concentrations in culture media supplemented with indicated EGTA concentrations, measured using the Ca^++^-sensitive electrode and the standard curve in panel B. Free Ca^++^ is presented as pCa = −log([Ca^++^]) in moles/L. Note the marked and nonlinear reductions in free Ca^++^ concentrations with increasing EGTA. In panels B and C, symbols represent mean of replicate measurements with error bars typically smaller than the symbols

Previous studies used addition of EGTA to standard culture medium to determine that *P. falciparum* lines require extracellular Ca^++^ for propagation [4–6]; EGTA toxicity from mechanisms other than chelation of divalent cations was excluded because equimolar addition of CaCl_2_ fully restores parasite growth. Here, we hypothesized that there may be reproducible differences in Ca^++^ requirement for parasite clones; such differences could be used to explore possible molecular targets and the precise roles served by Ca^++^. We therefore surveyed several laboratory clones and measured parasite growth inhibition by a range of EGTA concentrations (Fig. 1a). Under our experimental conditions, 0.45 mM EGTA effectively abolished expansion of cultures for each of the examined parasite lines. This concentration is consistent with stochiometric chelation of Ca^++^, which is present at a nominal 0.42 mM concentration in standard RPMI 1640 medium; addition of lipid-rich bovine albumin preparations, as required for parasite cultivation, may affect the free Ca^++^ available for EGTA chelation. Lower EGTA concentrations yielded survival and growth that differed significantly depending on parasite genotype (*P* < 0.01 for one-way ANOVA comparisons at 0.33, 0.36, and 0.39 mM EGTA, *n* = 3 to 7 trials each). At concentrations that produced incomplete growth inhibition, we observed that Pf803 and Dd2 were consistently the least and most resistant to EGTA addition, respectively.

While these growth inhibition experiments utilized relatively small changes in EGTA concentration, the free Ca^++^ concentration in the culture medium is expected to change dramatically and nonlinearly with incremental addition of chelator [25]. We therefore used a Ca^++^-sensitive electrode to estimate free Ca^++^ in culture media supplemented with EGTA. At concentrations up to 0.4 mM, EGTA addition reduced free Ca^++^ stoichiometrically; higher concentrations produced logarithmic reductions in free Ca^++^, but precise estimation was limited by electrode sensitivity (Fig. 1b and c). Because the Ca^++^ affinity of EGTA depends on temperature, pH and other factors, we performed these electrode measurements at 37 °C and attempted to simulate the conditions encountered in parasite culture. Our estimates may nevertheless be adversely affected by changes in pH under culture conditions due to metabolic acids produced by parasites and the presence of Ca^++^-chelating agents such as intracellular proteins and incompletely characterized metabolic byproducts. With these caveats in mind, the *IC*_*50*_ values for the parasite lines we examined, between 0.34 and 0.40 mM EGTA, correspond to half-maximal requirement (*EC*_*50*_) between 11 and 40 μM free extracellular Ca^++^ for the parasite lines we examined.

Each of the parasite clones in Fig. 1a has been used to generate one or more *P. falciparum* genetic crosses [26]. Thus, the differences in EGTA susceptibility in Fig. 1 could be examined in cross progeny to identify genetic loci involved in Ca^++^ requirement. Amongst the four available genetic crosses, the Dd2 x HB3 pair of parental lines exhibited the greatest difference in EGTA susceptibility and was, therefore, selected for further study.

### Inheritance in the Dd2 x HB3 cross

**Fig. 2:**
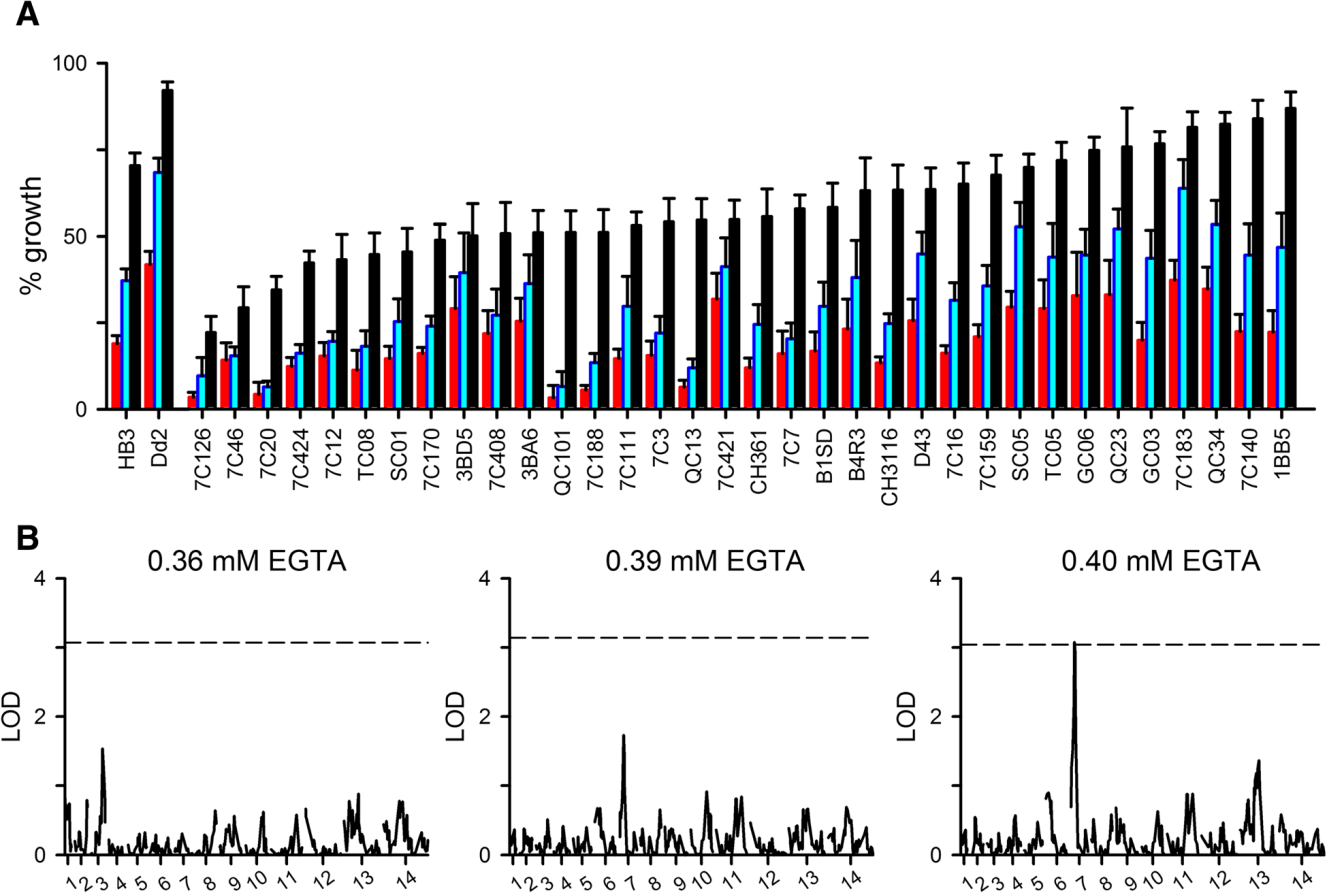
Complex inheritance of Ca^++^ requirement in the Dd2 x HB3 genetic cross. **a** Mean ± S.E.M. % growth values for the Dd2 and HB3 parental lines and the available progeny clones in the presence of 0.36, 0.39, and 0.40 mM EGTA (black, blue, and red bars, respectively), normalized to matched controls without EGTA. *n* = 49–53 for parental lines, and 7–17 trials for each progeny clone. Progeny clones are ordered according to increasing % growth in 0.36 mM EGTA. **b** Logarithm of odds (LOD) scores from a primary QTL scan for each EGTA concentration. In each panel, the dashed line represents the *P* = 0.05 significance threshold calculated from 1000 permutations

To search for genetic loci that may determine in vitro susceptibility, we performed EGTA growth inhibition studies with each of the 34 available independent progeny clones from the Dd2 x HB3 genetic cross. Each clone was examined in dose response experiments using 0.36, 0.39, and 0.40 mM EGTA and evaluated in 7–17 independent trials. For each trial, growth was normalized to 100% for matched controls grown without EGTA to correct for known differences in propagation rates for these lines [27]. Throughout these studies, we continued to evaluate EGTA sensitivities of Dd2 and HB3 in parallel with the progeny to evaluate stability of the parental phenotypes on continued cultivation and as controls for measurements using progeny clones. These studies confirmed stable differences between the parental lines (*P <* 10^− 3^ at 0.36, 0.39, and 0.4 mM EGTA, *n* = 49–53 independent trials) and revealed a range of EGTA sensitivities for the progeny clones (Fig. 2a), consistent with one or more genetic loci that determine in vitro Ca^++^ requirement. Many progeny clones were more sensitive to EGTA than either parent; some exhibited intermediate values. Because the progeny were ordered in Fig. 2a according to increasing tolerance to 0.36 mM EGTA (black bars), this plot also reveals that the relative sensitivities to 0.39 and 0.40 mM EGTA could not be confidently predicted by measurements at a single, lower concentration. For example, the QC101 and 3BA6 daughters expanded equally well with 0.36 mM EGTA, but 3BA6 tolerated the two higher EGTA concentrations better than QC101.

These observed differences in dose response steepness have several explanations [28]. Here, a conservative hypothesis is that Ca^++^ serves multiple roles over the course of the parasite cycle such as facilitating intracellular development, parasite egress and re-invasion. Each of these target sites may have a distinct dissociation constant, *K*_*d*_, for Ca^++^ binding. The overall shape of the EGTA dose response is then determined by summing the growth inhibitory effects of Ca^++^ removal at each target site based on the corresponding *K*_*d*_ values. Distinct parasite genomic loci presumably define these target sites and their *K*_*d*_ values. Thus, the observed range of EGTA susceptibilities and dose response profiles in the HB3 x Dd2 cross progeny are consistent with non-Mendelian or complex inheritance.

We then used QTL analysis to search for inherited genetic loci associated with growth at each EGTA concentration. We sought correlations between inheritance of mean growth at each EGTA concentration and each of 448 microsatellite markers from the 14 parasite chromosomes. This statistical approach identified a single locus that reached weak statistical significance at 0.4 mM EGTA (Fig. 2b, *P* = 0.05 for the 3E7 microsatellite marker on chromosome 7 at 0.4 mM EGTA). It also found a few minor peaks that did not reach statistical significance, including the 3E7 marker when growth was evaluated at 0.39 mM EGTA.

### No obvious candidate genes or association with haplotypes of PfCRT, the chloroquine resistance transporter

The chromosome 7 locus associated with the positive 3E7 microsatellite marker is flanked by two negative markers, HSP86 and 7A11. Because the polymorphism(s) that mediate differences in EGTA sensitivity could be situated anywhere between these negative markers, we examined this entire 45 kB region and found it contains 12 protein-coding genes and two putative small nucleolar RNAs. None of these candidates has conventional Ca^++^-binding domains or annotated roles that could readily account for changes in EGTA sensitivity.

Interestingly, this chromosome locus has previously been implicated in inheritance of resistance to chloroquine, once the mainstay of antimalarial chemotherapy. Molecular studies have implicated mutations in PfCRT, a polytopic membrane protein at the parasite’s digestive vacuole [29]. While it is clear that these mutations confer chloroquine resistance, whether this protein is a transporter and its physiological role remain debated; in addition to direct efflux of chloroquine via PfCRT, hypotheses include roles in transport of H^+^ [30], amino acids [31], iron [32], glutathione and other organic solutes [33, 34], Cl^−^ [35], and Ca^++^ [36]. Our observation that tolerance to EGTA may also be partially determined by this locus suggests a role in Ca^++^ transport for PfCRT or other gene products in the mapped locus.

**Fig. 3:**
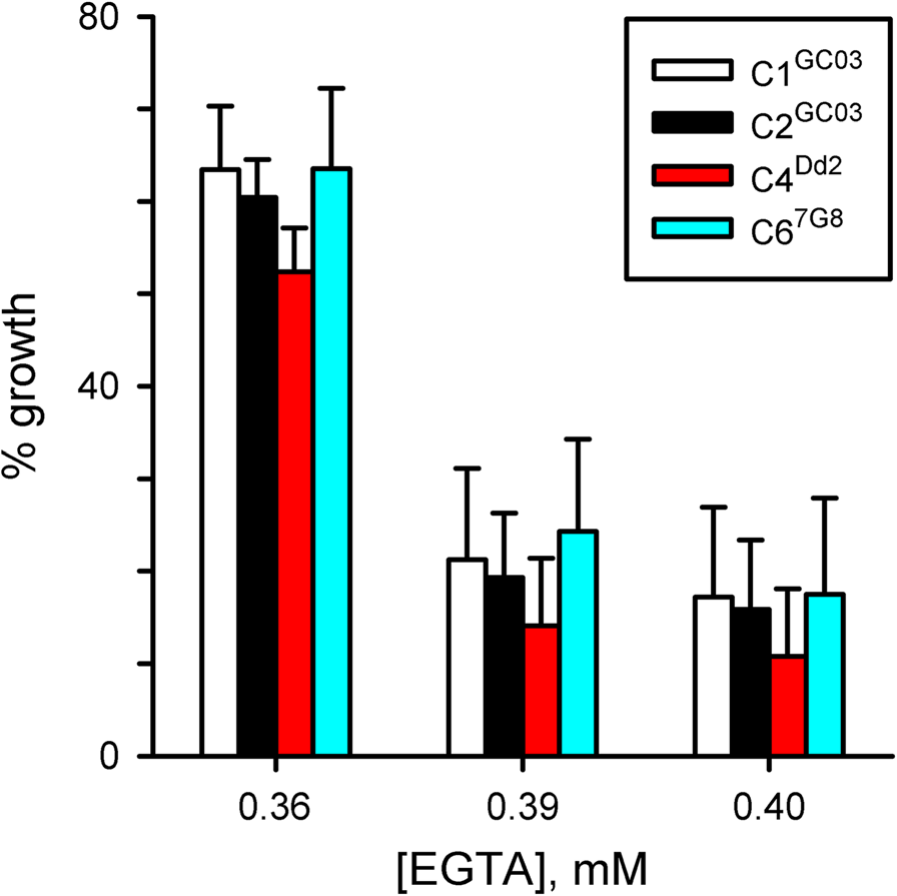
PfCRT haplotypes do not directly influence parasite Ca^++^ requirement. Mean ± S.E.M. normalized growth for indicated transfectant lines at each EGTA concentration. *n* = 10–11 for each line. Neither parasite transfection nor altering PfCRT haplotype have a significant effect on EGTA susceptibility (*P* > 0.1 in ANOVA comparisons)

To test this possibility, we performed EGTA growth inhibition dose response experiments with the C1^GC03^, C2^GC03^, C4^Dd2^, and C6^7G8^ transfectant lines [29]. These lines were generated in the GC03 chloroquine sensitive progeny clone from the Dd2 x HB3 cross and designed to confer resistant PfCRT haplotypes (C4^Dd2^ and C6^7G8^) or serve as transfection controls (C1^GC03^ and C2^GC03^) for possible effects of transfection or the associated *hDHFR* and *BSD* selectable markers (human dihydrofolate reductase and blasticidin S deaminase, respectively). EGTA dose response studies with these lines revealed no significant differences between these four transfectants (Fig. 3); there were also insignificant effects of transfection or selectable markers (*P >* 0.1 at each EGTA concentration for one-way ANOVA comparisons amongst the four transfectant lines and in comparisons of the two transfection controls with wild-type GC03, *n* = 10 or 11 trials for each transfectant line). These experiments suggest that PfCRT haplotype does not influence Ca^++^ utilization in the intracellular parasite. As other genomic loci may interact with this locus and these transfections may not yeild the maximally effective haplotypes, additional studies including transfections that do not use multiple selectable markers may be required to determine whether PfCRT or other genes in this locus contribute to parasite Ca^++^ requirement.

How might this locus determine variations in Ca^++^ requirement in the HB3 x Dd2 genetic cross? Although the absence of Ca^++^-binding domains in any of the 12 protein-coding genes in this locus may be discouraging, we recognize that *Plasmodium spp.* may use proteins without clear orthologs in other systems to serve critical roles. A good example is the *clag* gene family [37], which is conserved in all examined *Plasmodium spp.* but absent from other genera. Although the encoded CLAG proteins do not resemble ion channel proteins, genetic mapping and DNA transfection implicated a primary role for this gene family in the plasmodial surface anion channel (PSAC), a nutrient uptake channel at the host erythrocyte membrane [12]. As used in that study, an agnostic approach to identifying the gene(s) that govern Ca^++^ requirement would involve allelic exchange DNA transfection for each of the 12 candidates in the 3E7 marker-associated locus; EGTA dose response studies as we have performed here could then uncover the responsible gene(s). Ongoing improvements in parasite DNA transfection technology may make this a less effort- and time-consuming approach.

### Ca^++^ requirements during merozoite egress and invasion

**Fig. 4:**
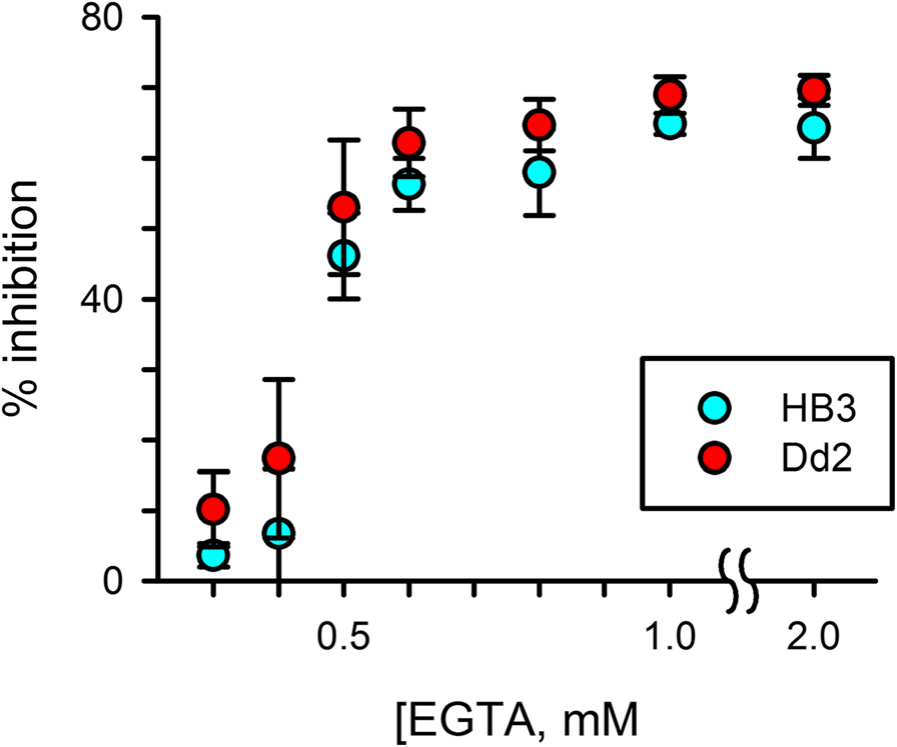
The extracellular Ca^++^ requirements for merozoite egress and reinvasion are indistinguishable in the Dd2 and HB3 parental lines (red and blue symbols, respectively). Mean ± S.E.M. reduction in ring-stage parasitemia after treatment of schizont-stage cultures with indicated EGTA concentrations, normalized to 100% for matched treatment with 5 μM cytochalasin D

Two of the most critical events that require Ca^++^ are the egress of daughter merozoites at the end of the intracellular cycle and the subsequent reinvasion of new erythrocytes to form ring-stage parasites. Egress is dependent on intracellular Ca^++^ and does not appear to require extracellular Ca^++^ [14, 38]; in contrast, reinvasion is thought to require primarily extracellular Ca^++^ [39]. We therefore examined whether Ca^++^ requirements during egress and reinvasion differ between Dd2 and HB3 parasites. Experiments using a short ~ 5 h application of EGTA to tightly synchronized late-stage schizonts followed by microscopic examination and quantification of ring-stage parasitemias with flow cytometry revealed dose-dependent reduction in successful progression to rings for both Dd2 and HB3 (Fig. 4), with inhibition plateaus at 65–70% of those seen with the positive control cytochalasin D. The EGTA dose responses for Dd2 and HB3 were indistinguishable, with *IC*_*50*_ values of 0.57 mM ± 0.04 for both lines (*P* 0.31, *n* = 3 independent trials); these values were higher that observed in our standard growth inhibition experiments, as might have been predicted given the shorter duration of EGTA application. Thus, differing Ca^++^ requirements for egress or reinvasion do not account for the difference in EGTA sensitivities of these parasite lines.

## Discussion

Calcium serves many roles in cells, including control of cell cycle regulation, DNA replication, modulation of enzymes, and signaling [40]. Over the course of their asexual bloodstream cycle, malaria parasites undergo each of these activities. Nevertheless, the roles served by Ca^++^ in these and other cellular processes unique to these pathogens such as host cell egress and invasion or ion homeostasis within specialized organelles have been difficult to study for several reasons. The multiple compartments within infected cells make precise quantification of Ca^++^ at target sites difficult. Manipulation of free Ca^++^ concentrations at individual target sites is also currently not feasible. The low Ca^++^ concentration within the host cell, maintained by an Ca^++^ ATPase extrusion pump at the erythrocyte surface, also complicates basic studies aimed at mechanistic insights. Finally, transient changes in Ca^++^ concentrations, as observed and tracked in other cells [40, 41], are more difficult to detect within infected erythrocytes because of their small size and quenching by host cell hemoglobin. While Ca^++^ transients and spatial gradients have been well-studied in larger muscle cells and neurons [42, 43], the small compartments within infected cells require measurements near or beyond the limit of spatial resolution in conventional confocal microscopy.

To overcome these limitations, our study used the Ca^++^ chelator EGTA to determine that *P. falciparum* lines exhibit stable differences in extracellular Ca^++^ requirement for in vitro cultivation. We then tracked this difference in the progeny clones of the Dd2 x HB3 genetic cross; this approach revealed stable, heritable differences in parasite Ca^++^ requirement that may be defined by multiple genetic loci. A key limitation we encountered was the relatively small range of EGTA concentrations over which differences between Dd2 and HB3 are observed. This narrow range is determined primarily by exponential decreases in free Ca^++^ concentrations resulting from further addition of EGTA once the total Ca^++^ and EGTA concentrations are approximately equal [25]. The steep reduction in parasite survival with small increases in EGTA may also reflect synergistic killing due to action on multiple parasite Ca^++^-dependent targets. While these differences in EGTA tolerance were modest, their statistical significance provides a framework that may eventually lead to identification of key molecular determinants. For example, a modest difference between parental lines for PSAC inhibition by furosemide in the 3D7xHB3 cross failed to detect significant QTL [44]. Although unsuccessful in finding the genes, that study stimulated high-throughput screens to find a Dd2-specific inhibitor and enabled successful genetic mapping to implicate *clag3* genes in PSAC formation [12, 23].

Complex inheritance is consistent with multiple targets for Ca^++^, which may act at various developmental points in the 72 h growth inhibition assays we used. The observed clone-dependent differences in EGTA susceptibility presumably reflect polymorphisms in key target proteins. Polymorphisms in transporters or Ca^++^-dependent enzymes may directly lead to altered Ca^++^ affinities or may indirectly affect growth if the polymorphisms affect enzyme kinetics. Heritable differences could also reflect polymorphisms in proteins that do not bind Ca^++^; one such possibility is a Ca^++^-independent transcription factor activated by Ca^++^-dependent phosphatases to then control cell cycle progression [45]. The stable differences in progeny clone phenotypes shown in Fig. 2 could also reflect stable epigenetic marks under in vitro culture conditions rather than heritable genome-level differences, as exemplified by epigenetic regulation of *var* and *clag3* genes in malaria [46, 47].

The potential locus we identified on chromosome 7 is particularly interesting as it encodes PfCRT, a putative transporter implicated in clinical resistance to chloroquine and other antimalarial drugs [29]. Notably, several studies have proposed Ca^++^ affects chloroquine action and digestive vacuolar physiology, based primarily on the effects of Ca^++^ channel inhibitors and calmodulin antagonists [48, 49]. Our growth inhibition studies with transfectant lines carrying modified *pfcrt* alleles did not alter EGTA sensitivity. Although this result does not itself support a link between Ca^++^ availability and PfCRT activity, it is possible that additional transfections using different genetic backgrounds and other polymorphisms may confirm the proposed roles of Ca^++^ in chloroquine susceptibility. Alternatively, one or more of the other genes within the mapped locus may encode Ca^++^-dependent activities. As linkage analysis produced only a marginally significant QTL, it will be important to have greater confidence in this locus, possibly though identification and characterization of a larger number of progeny clones. Studies that examine how Ca^++^ concentration affects digestive vacuolar physiology may also be complicated by Ca^++^ indicator dye import via PfMDR1 (*P. falciparum* multidrug resistance gene product), another transporter on this vacuole [50].

Our studies have identified differences in Ca^++^ utilization by *P. falciparum* parasite lines. These differences may reflect variable requirement amongst parasite clones in human infections to confer survival advantage under stress conditions, such as hypocalcemia associated with malnutrition [51]. Alternatively, they may have arisen through selective pressures during adaptation to in vitro culture. In either case, the observed differences and unbiased studies such as these linkage analyses have the potential to uncover Ca^++^-dependent processes as targets for therapy development.

## Methods

### Parasite cultures

*Plasmodium falciparum* laboratory lines were cultivated separately in RPMI 1640 with L-glutamine (Thermo Fisher Scientific, Waltham, MA) supplemented with 50 mg/L hypoxanthine, 31 mM NaHCO_3_, 25 mM HEPES, 10 μg/mL gentamicin, and 0.5% NZ microbiological BSA (MP Biomedicals, Santa Ana, CA) using O^+^ human erythrocytes obtained from anonymous donors (VA Blood Services, Richmond, VA). Cultures were maintained at 5% hematocrit under 5% O_2_, 5% CO_2_, 90% N_2_ at 37 °C. Parasite genotypes were confirmed with molecular studies.

### Growth inhibition assays

Parasite Ca^++^ requirement for in vitro propagation was evaluated by addition of EGTA to culture media; experiments were identically performed for all laboratory parasite lines and progeny clones. A standard 72 h growth assay and the SYBR Green I dye were used to measure parasite nucleic acid production, as described previously [6]. Parasite cultures were synchronized with 5% sorbitol prior to each trial. These ring-stage cultures were seeded in 96-well microplates at 2% hematocrit and 0.5–1.0% parasitemia in the above culture medium with indicated EGTA concentrations. pH measurements were used to ensure that EGTA addition did not change the pH of the medium. Microplate cultures were then incubated at 37 °C under 5% O_2_, 5% CO_2_, 90% N_2_ without medium changes. Expansion of cultures during this incubation was evaluated by addition of lysis buffer (20 mM Tris, 10 mM EDTA, 0.016% saponin, 1.6% triton X100, pH 7.5) with SYBR Green I nucleic acid gel stain at a 5000x dilution (Invitrogen, Carlsbad CA). After a 45 min incubation without ambient light, fluorescence measurements (excitation/emission wavelengths of 485 nm and 528 nm) were used to quantify parasite DNA. Growth was calculated as the mean of triplicate wells after subtracting background fluorescence from matched cultures seeded with 20 μM chloroquine to abolish parasite expansion. These values were then normalized to 100% for matched cultures grown without chelator or inhibitor. Control experiments with stoichiometric addition of CaCl_2_ to EGTA confirmed that growth is fully restored, excluding nonspecific EGTA toxicity.

### Free [ca^++^] measurements

Free Ca^++^ concentrations in culture media with and without EGTA addition were measured with an ion sensitive electrode (Cole-Parmer, Vernon Hills, IL). Media were gassed and measured at 37 °C to match pH and temperature encountered by parasites under in vitro culture. Freshly prepared Ca^++^ standards were also measured at 37 °C and used to estimate an electrode slope of 26.7 mV/decade, indicating electrode specificity for Ca^++^. Media and standards were measured after addition of 4 M KCl as an ionic strength adjuster, according to the electrode manufacturer.

### Linkage analysis

QTL analysis to search for parasite genomic loci involved in Ca^++^ utilization was performed as described [24]. We used 448 previously selected microsatellite markers that distinguish the Dd2 and HB3 parasite lines and define the available progeny clones from this genetic cross [52]; the full list of markers and their sequences are available at https://www.ncbi.nlm.nih.gov/probe/?term=10558988[pmid]. QTL analysis was performed by the multiple imputation method [53] as implemented in the R/qtl software (freely available at http://www.rqtl.org/) [54]. A *P* = 0.05 significance threshold, shown as a dashed line in each QTL genome scan, was determined through analysis of 1000 permutations.

### Merozoite egress and reinvasion assays

EGTA dose response studies were used to examine the Ca^++^ requirement during egress and reinvasion at the end of the asexual parasite lifecycle using modifications to a previously described method [55]. Dd2 and HB3 parasite lines were tightly synchronized with Percoll/sorbitol enrichment of schizont-stage cultures, cultivation with fresh erythrocytes for 6 h, and a 5% sorbitol treatment. Subsequently, serial Giemsa-stained smears were performed until a majority of mature schizonts were observed, typically after 38–40 h. Synchronized schizonts were again enriched with Percoll/sorbitol, added to fresh erythrocytes, and resuspended in 96-well microplates at a 1% hematocrit and 2% parasitemia with complete medium and indicated EGTA concentrations. These parasites were cultivated at 37 °C with 5% CO_2_ and regular monitoring until the ring-stage parasitemia reached 2%, approximately 5 h. 20 μL of each suspension culture was then transferred to a fresh microplate with 80 μL of 1X SYBR Green I in 1x PBS, incubated for 30 min at room temperature, and washed twice in 1x PBS. The stained cells were resuspended in 100 μL of 1x PBS and the number of ring-infected erythrocytes counted using an Accuri C6 flow cytometer (BD Biosciences, Franklin Lakes, NJ). The data were normalized to percent inhibition of invasion using two matched controls (no inhibitor and 5 μM cytochalasin D) present in each microplate. *IC*_*50*_ values were calculated in SigmaPlot 10 (Systat Software, San Jose, CA).

### Statistical methods

Statistical significance was evaluated using Student’s *t* tests or one-way analysis of variance (ANOVA) as indicated, using SigmaPlot 10 or Prism 7 software.

## Abbreviations

*BSD*: Blasticidin S deaminase gene
*clag* and CLAG: Cytoadherence linked antigen gene and protein, respectively
EGTA: Ethylene glycol-bis(β-aminoethyl ether)-N,N,N′,N′-tetraacetic acid)
*hDHFR*: Human dihydrofolate reductase gene
*IC*_*50*_: Half-maximal inhibitory concentration
*K*_*d*_: Dissociation constant
PfCRT: *P. falciparum* chloroquine resistance transporter
PfMDR1: *P. falciparum* multidrug resistance gene product
PSAC: Plasmodial surface anion channel
QTL: Quantitative trait locus
